# Varicose Veins

**Published:** 1892-10-29

**Authors:** 


### GLASGOW WESTERN INFIRMARY.

### Varicose Veins.

The following is a brief summary of the methods usually employed in the Glasgow hospitals in the treatment of this troublesome affection. Necessarily the treatment falls under two heads: 1st, Palliative; 2nd, Curative.

Of the palliative measures, by far the most general is the use of Martin's bandage, a pure rubber bandage, two and a half inches broad and from four to six yards in length, which, when properly applied, supports the relaxed walls of the veins and to a great degree relieves the pain and discomfort of the distension which follows a prolonged upright position. Judiciously used also, especially if aided by such tonic measures as a morning and evening cold bath and massage, there may even follow a partial restoration to the normal lumen of the vessels.

In applying the bandage, however, there are certain points to be attended to, without which nothing but disappointment will be met with.

1st. The bandage ought to be kept scrupulously clean, best sponged with plenty of cold water when taken off at bedtime, and hung up to dry in a place which may be reached without the patient getting out of bed.

2nd. It must be put on before the patient assumes the erect attitude, while yet the veins are undistended and œdema absent, so that in assuming the erect posture neither of these may occur.

3rd. The bandage must be uniformly applied, not tightly, but with sufficient pressure to lie smoothly. If put on tight, then the tension becomes unbearable; when the patient gets up the bandage is slackened away, or taken off and reapplied, and the first principle broken.

4th. The skin must be bathed morning and evening, and dusted with Fuller's Earth, or other powder.

5th. The bandage is not to be worn when the patient is in bed, as if worn constantly the skin under the impervious covering tends to become eczematous, and so renders the use of the bandage impossible.

The bandage is cheap and durable, and on the whole eminently useful. The principal objection to its use is the discomfort following cutaneous irritation and improper application. The first of these objections may be partly got over by use of a bandage with numerous circular perforations, allowing the perspiration to escape, but more especially by absolute cleanliness. Elastic stockings are little used, and necessarily, from their expense and want of durability, are only available to the rich, and unless very carefully made to measure are apt to do more harm than good, as frequently the upper edge of the stocking is stronger than the rest, which, yielding, allows the blood to accumulate, and then the upper border acts the part of a moderately firm ligature, with its constant evil effects on the return course of the blood.

The ordinary calico bandage carefully applied before the limb becomes engorged with blood gives a fair amount of relief, but difficulty is generally experienced in teaching patients its proper application, so that the bandage may remain in position while the patient pursues his usual avocation.

Needless to say that with such external support to the relaxed veins must ever be associated a careful treatment as far as ascertainable of the causes, whether direct or predisposing, a careful attention to the condition of the bowels, the presence of tumours or other accumulations in the pelvis, the use of that abomination, the garter, in the female, or any other thing which may interfere with the return course of the blood; also the correction of dietetic errors and constitutional diathesis.

Radical treatment for the most part does not consist so much in an attempt to cure the diseased vein as to extirpate it, either in its entirety or to destroy its functions by ligature. Excepting a method which has gained favour lately with some, viz.:

First, blistering and rest. The blister is put over the course of the vein for a distance of about three or four inches, with a width overlapping the vein on both sides about a quarter of an inch. The Liq. Epispasticus applied with a brush to a carefully cleaned skin acts admirably. The irritation set up by the blister induces a healthy reaction in the vein wall, which must never during the treatment be distended with blood, and also in the peri-vascular space an inflammatory deposit and condensation fixing the vein in its more or less collapsed condition. The blistering is repeated and applied to one part after another until the whole of the affected veins have been got over twice or even three times. The horizontal position is a *sine qua non*, and this not intermittently, but during the whole course of treatment, which usually lasts for from two to three months, and to those who will consent to the somewhat trying ordeal it affords good hope of what most nearly approximates to a real cure of varicose veins.

*Operative Interference.*—Excision of varicose veins is now frequently practised, and, although some surgeons taboo the operation entirely, there is no doubt but that great relief follows the complete removal of the affected vein.

The operation is easily performed if the veins are first distended by a lightly applied tourniquet, so that they may be rendered more apparent, and care taken during the dissection to prevent the escape of the blood by free use of the ligature. Not only is the operation easy, but with the usual antiseptics, free from danger and rapidly recovered from, so that patients are usually back to work within fourteen days.

The operation is carried out as follows: The skin is cleansed with soap and water, afterwards washed with turpentine, and this followed by methylated spirits, and 1 in 20 carbolic, or 1 in 1,000 solution of perchloride of mercury. The veins being engorged with blood, an Esmarch's band, or better, several turns of an elastic bandage is applied to arrest the circulation. An incision is then made over the affected vein, corresponding in length to the extent of the vein to be removed, usually determined by the external appearance of swelling. Occasionally the incision extends from the upper end of the lower third of the femur to opposite a corresponding point on the tibia, the vein usually affected being the internal saphenous, though frequently the external as well.

The vein having been exposed the branches are ligatured, the object being to retain the blood in the affected vein, rendering its removal easier and more accurate. The tourniquet removed and hemorrhage arrested, the skin wound is accurately stitched. As a rule no drainage tube is required, and under a dry dressing, the wound will be found healed in a week or ten days.

The excision of the affected veins as a rule gives relief, the venous circulation being carried on by the deep veins, which, supported by the muscles, have less tendency to dilate.

*Subcutaneous Ligature.*—An operation sufficiently easy to perform but less certain in its results. It is less frequently practised, as it is found that the branches soon form a collateral circulation communicating with the dilated portions of the vein and thus perpetuating the evil.

Intra-venous injections are not now practised.

